# Unusual structural and electronic properties of porous silicene and germanene: insights from first-principles calculations

**DOI:** 10.1186/s11671-014-0704-3

**Published:** 2015-01-27

**Authors:** Yi Ding, Yanli Wang

**Affiliations:** Department of Physics, Hangzhou Normal University, 16 Xuelin Street, Hangzhou, 310036 People’s Republic of China; Department of Physics, Center for Optoelectronics Materials and Devices, Zhejiang Sci-Tech University, 5 Second Avenue, Xiasha Higher Education Zone, Hangzhou, 310018 People’s Republic of China

**Keywords:** Si/Ge nanosheet, Tunable bandgap, Solar energy application

## Abstract

Using first-principles calculations, we investigate the geometric structures and electronic properties of porous silicene and germanene nanosheets, which are the Si and Ge analogues of *α*−graphyne (referred to as silicyne and germanyne). It is found that the elemental silicyne and germanyne sheets are energetically unfavourable. However, after the C-substitution, the hybrid graphyne-like sheets (c-silicyne/c-germanyne) possess robust energetic and dynamical stabilities. Different from silicene and germanene, c-silicyne is a flat sheet, and c-germanyne is buckled with a distinct half-hilled conformation. Such asymmetric buckling structure causes the semiconducting behaviour into c-germanyne. While in c-silicyne, the semimetallic Dirac-like property is kept at the nonmagnetic state, but a spontaneous antiferromagnetism produces the massive Dirac fermions and opens a sizeable gap between Dirac cones. A tensile strain can further enhance the antiferromagnetism, which also linearly modulates the gap value without altering the direct-bandgap feature. Through strain engineering, c-silicyne can form a type-II band alignment with the MoS _2_ sheet. The combined c-silicyne/MoS _2_ nanostructure has a high power conversion efficiency beyond 20% for photovoltaic solar cells, enabling a fascinating utilization in the fields of solar energy and nano-devices.

## Background

Since the discovery of graphene, two-dimensional (2D) carbon nanostructures, due to their superior physical properties, have attracted substantial concerns from the fields of nano-sciences and nano-materials [1-3]. As a 2D carbon nanosheet, graphene is made of *sp*^2^-hybridized C atoms that are regularly arranged in a honeycomb lattice. The *p*_*z*_ orbitals of C atoms are half-filled in graphene, which results in the Dirac-like electronic structure with a semimetallic feature [2,4]. Derived from graphene, several 2D carbon allotropes have been proposed [5]. Among them, a porous carbon sheet called graphyne, which is made of both *sp*^2^- and *sp*-hybridized C atoms, becomes a rising star on the horizon of graphene-related studies [6,7]. In the graphyne sheet, *sp*-hybridized C atoms form acetylene bonds to link the *sp*^2^-hybridized ones. Depending on the ratio of *sp* constitute, graphyne can be generally classified into the *α*, *β* and *γ* types [8]. In the *α* type, each acetylene bond links one *sp*^2^-hybridized C atom, while in the *β* and *γ* types, it links a pair and a hexagon of *sp*^2^-hybridized C atoms, respectively [8-11]. The *α* type (*α*-graphyne) is a representative system for graphyne, which keeps the same hexagonal symmetry as graphene [12-16]. Alpha-graphyne can be regarded as an amplified graphene by inserting additional acetylene bonds into the place of C-C bonds. The semimetallic behaviour is still presented in the sheet [12-15], and the corresponding one-dimensional graphyne nanoribbons also possess similar electronic properties to the graphene ones [15,16]. Besides these common types of graphyne, theoretical studies also predict the existence of other possible structures, such as graphdiyne [17,18], 6,6,12-graphyne [10,19], *δ*-graphyne [20] and so on. In the experiments, graphdiyne and *γ*−graphyne have been synthesized through the metal-catalyzed cross-coupling reaction [21,22]. The graphdiyne-based nanotubes and nanowires have also been fabricated by the special templated synthesis method [23,24]. Experimenters further find the doping of graphdiyne benefits the photoconversion processes, which raises the power conversion efficiency of solar cells [25,26].

Very recently, silicene and germanene nanosheets, which are the Si and Ge counterparts of graphene, have been produced in the experiments [27,28]. Different from graphene, the basal planes of silicene and germanene are buckled with a chair-like buckled conformation [29]. The Dirac-like electronic structures are still present in these buckled sheets [30]. A lots of investigations have been performed on silicene and germanene [31-45], which are found to possess several peculiar characteristics, such as electric-field/substrate-induced gaps [31-35], strain-induced self-dopings [36-39], high-efficient thermoelectric performances [40-42], and promising applications for Li batteries [43-45]. However, so far, the information about the porous silicene and germanene, especially the graphyne-like Si and Ge ones (which we refer to as silicyne and germanyne thereafter), is still unknown. What are favourable structures for these porous sheets? How about their stability comparing to other 2D Si/Ge nanosheets? Do they possess particular electronic properties? To address these issues, we perform a comprehensive first-principles investigation on silicyne and germanyne, for which the unusual atomic structures and electronic properties are revealed in detail.

## Methods

The first-principles calculations are performed by the VASP code with projector augmented wave pseudopotentials and plane-wave basis sets [46,47]. The cutoff energy of plane-wave basis is set to 500 eV, and a vacuum layer up to 15 Å is utilized to simulate the isolated 2D sheet. The Monkhorst-Pack scheme is adopted to sample the Brillouin zone, which uses a 9×9×1 and 15×15×1**k**-mesh in the relaxed and static calculations, respectively. During the structural relaxation, the Perdew-BurkeErnzerhof (PBE) exchange and correlation (XC) functional are used. All the lattice constants as well as atomic coordinates are fully optimized until the convergence of force on each atom is less than 0.01 eV/Å. The hybrid XC functional of Heyd-Scuseria-Ernzerhof (HSE) is also used in the band structure calculations, which adopts the HSE06 form with a screening parameter of 0.11 bohr ^−1^ [48]. In view of the large computing resources required for the hybrid functional, HSE calculations are performed by the FHI-aims code with a numeric local orbital basis set [49]. We find that the two codes give consistent results despite of the basis set difference. The dynamical stabilities of nanosheets are studied by the Phonopy code [50], which is performed on a supercell with 4×4 units.

## Results and discussion

### Structural stability

**Figure 1:**
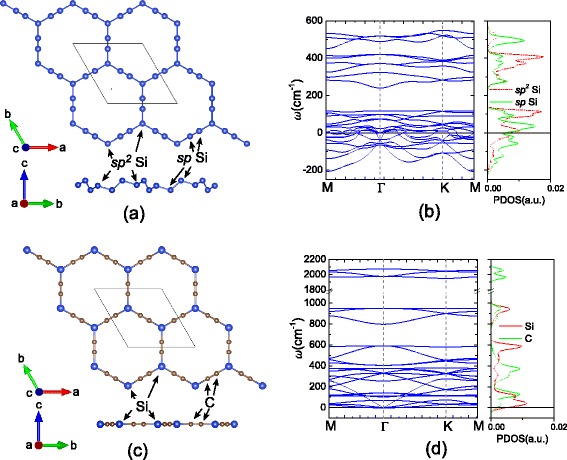
**Structures and phonon dispersion curves.** The optimized structures of **(a)** elemental and **(b)** C-substituted silicyne sheets, **(c)** and **(d)** the corresponding phonon dispersion curves and densities of states.

Firstly, the elemental silicyne sheet is investigated. After full structural optimization, the Si atoms are buckled in silicyne as shown in Figure 1a. In this buckled structure, the twofold-coordinated Si atoms have a higher buckling height than the threefold-coordinated ones. The cohesive energy (*E*_coh_), defined as the difference between the energy of compound and the sum of the corresponding isolated atomic energies, is −3.11 eV per atom for silicyne. As listed in Table 1, this value is larger than that of silicene (−3.95 eV) and much higher than the graphyne value (−7.03 eV), suggesting the weaker stability of elemental silicyne. Figure 1b depicts the phonon dispersion of silicyne, which shows an amount of negative frequencies in the reciprocal space. This indicates the elemental silicyne sheet is dynamically unstable, which can not sustain the free-standing state. By analysing the partial density of phonon states, we find the negative frequencies are predominantly related to the twofold-coordinated Si atoms in the porous sheet. Thus, in order to obtain a stable structure, acetylene bonds are needed to replace the twofold-coordinated Si ones.

**Table 1:** **The cohesive energies of different nanosheets in the unit of eV per atom**

**Nanosheets**	**Energies**
Graphene	−7.95
Graphyne	−7.03
Silicene	−3.95
Silicyne	−3.11
Germanene	−2.60
SiC nanosheet	−5.97
c-Silicyne	−6.09
GeC	−4.91
c-Germanyne	−5.52^*a*^/ −5.55^*b*^

^*a*^Flat conformation and ^*b*^half-hilled one.

The C-substituted silicyne (c-silicyne) can be formed by inserting −*C*≡*C*− (acetylene part) into the place of Si-Si bonds of silicene sheet, as shown in Figure 1c. In the calculations, a buckled sheet is initially set for c-silicyne, but the optimized structure becomes a flat plane akin to graphyne. The C-C distance in c-silicyne is 1.24 Å, which is a typical value for the C-C triple bond. The Si-C distance is 1.76 Å, also close to the Si-C bond length in a hexagonal SiC sheet. As shown in Table 1, the cohesive energy of c-silicyne is −6.09 eV, which is about 3 eV smaller than that of elemental silicyne and even lower than that of the SiC sheet (−5.97 eV). Comparing to graphyne (−7.03 eV), the discrepancy in the cohesive energy stems from the bond difference between c-silicyne and graphyne. In the primitive cell of c-silicyne, it contains six Si-C bonds and three C ≡C bonds, while in graphyne the C-C bonds replace the Si-C ones. The energy difference between C-C and Si-C bonds (*ΔE*_CC−SiC_) can be evaluated from the cohesive energy difference between graphene and SiC sheets, which is 3/2*ΔE*_CC−SiC_=*E*_coh_(graphene)−*E*_coh_(SiC)=−1.98 eV. Based on this bond difference, the discrepancy of cohesive energy between graphyne and c-silicyne would be *E*_coh_(graphyne)−*E*_coh_(c−silicyne)=3/4*ΔE*_CC−SiC_=−0.99 eV, which agrees well with the first-principles result of −0.94 eV. Thus, owing to the C-substitution, the weaker Si-Si and Si ≡Si bonds are replaced by the Si-C and C ≡C ones, which greatly enhances the energetic stability of silicyne. Moreover, the phonon calculations in Figure 1d show that the c-silicyne sheet has no soft modes as a dynamically stable system. The good energetic and dynamical stabilities imply that the c-silicyne could be possibly synthesized experimentally. It is found that the acetylene part in c-silicyne induces high frequencies around the 2,000 cm ^−1^. These modes are from the stretching vibration of C ≡C bonds, which would be a Raman signature for the finding of c-silicyne [51,52].

**Figure 2:**
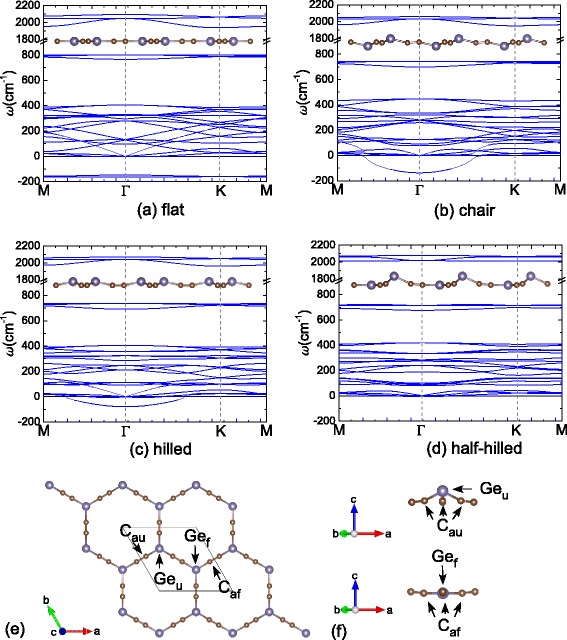
**The phonon dispersion curves of different conformations for c-germanyne (a-d).** The insets depict the lateral view for each conformation. **(e)** The top view of the half-hilled conformation. **(f)** Different local structures of Ge atoms in the half-hilled conformation.

**Figure 3:**
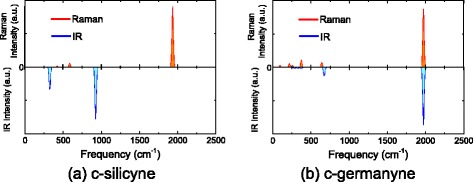
**The simulated Raman and IR spectra of (a) c-silicyne and (b) c-germanyne sheets.** A uniform Gaussian broadening of 10 cm ^−1^ is adopted in the plots of spectra.

For the germanyne, similar to the Si case, the elemental sheet has a large cohesive energy of −2.60 eV, which is higher than that of hexagonal germanene (−3.24 eV). The cohesive energy could be markedly reduced by the C-substitution, which becomes −5.52 eV for the c-germanyne with a flat conformation. This value is even lower than the GeC sheet as listed in Table 1. However, the flat conformation is dynamically unstable with a negative frequency about −150 cm ^−1^ in the reciprocal space as shown in Figure 2a. It suggests that a spontaneous buckle would arise in c-germanyne. To this end, a chair conformation is checked firstly, in which one Ge atom is upward and the other one is downward as shown in Figure 2b. After optimization, the chair conformation is 157 meV/unit lower than the flat one, but it is still dynamically unstable with a negative frequency of −136 cm ^−1^ at the *Γ* point. Then, a hilled-buckling structure is considered, for which the two Ge atoms in the unit cell are both upward as shown in Figure 2c. It is found that the hilled conformation is 16 meV/unit lower than the chair one and the soft mode is also weakened, whose negative frequency is reduced to −78 cm ^−1^. Finally, a half-hilled conformation is studied. As depicted in Figure 2d,e,f, in the unit cell, one Ge atom is upward while the other one locates in the same plane with neighbouring C atoms. The buckling height of the upshifted Ge atom (Ge _*u*_) is 0.94 Å, which pulls the three adjacent C atoms (C _au_) out of plane by about 0.12 Å. Such half-hilled conformation is dynamically stable with no soft modes as shown in Figure 2d. Furthermore, it has the lowest total energy for c-germanyne, which is 112, 128 and 235 meV/unit lower than the hilled, chair and flat ones, respectively. With the half-hilled conformation, the c-germanyne has a cohesive energy of −5.55 eV, which is 1.48 eV higher than the graphyne value due to the energy difference between Ge-C and C-C bonds. However, compared to the hexagonal germanene sheet (−3.24 eV), the c-germanyne has a stronger energetic stability, and thus, it is also much possible to be fabricated in the experiment. Figure 3 depicts the simulated Raman and infrared (IR) spectra of c-silicyne and c-germanyne by the PHonon of PWscf code [53]. It can be seen that a remarkable Raman peak is at about 2,000 cm ^−1^ for both sheets. This Raman signature is the fingerprint of graphyne-like structure [51,52], which would be also used to find the c-silicyne and c-germanyne structures. Besides that, due to the different buckling structures, the c-silicyne and c-germanyne have distinct IR spectra. The c-silicyne sheet has two noticeable IR peaks at the approximately 300 and 900 cm ^−1^, while the c-germanyne sheet has only one remarkable IR peak at the approximately 2,000 cm ^−1^. Thus, in the experiment, the Raman measurement can be used to identify the graphyne-like Si and Ge nanosheets, while the IR measurement can distinguish different buckling structures in them.

### Electronic structures

**Figure 4:**
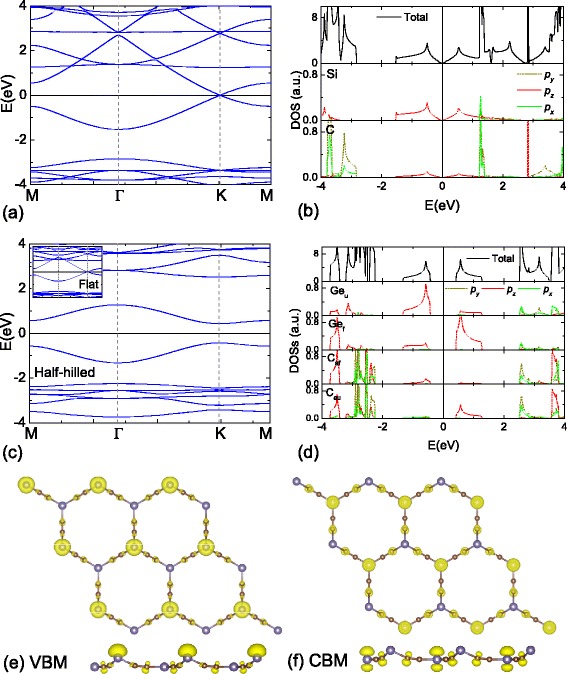
**The PBE band structures and DOSs for (a,b) c-silicyne and (c,d) c-germanyne.** The inset in **(c)** is the band structures of c-germanyne with the flat conformation. **(e,f)** The partial charge densities of VBM and CBM in c-germanyne. The isosurface is 0.1 e/Å ^3^.

The PBE band structures and density of states (DOSs) of c-silicyne are depicted in Figure 4a,b. Akin to graphyne, c-silicyne possesses a Dirac-like electronic property, for which the bottom conduction and top valence bands touch each other at the **K** point, i.e. Dirac point. The linear dispersions of bands are obtained around the Dirac point as $E(k)=\pm \hbar k v_{F}$, and the Fermi velocity *v*_*F*_ is estimated to be 5.4×10^5^ m/s by the PBE calculation. From the analysis of partial DOSs (PDOSs), it can be seen that the states around the Fermi level are dominated by the Si *p*_*z*_ orbitals. The C *p*_*z*_ orbitals also give a small contribution to these states. Some sharp *δ*-like peaks are evident at the −3.7, 1.2 and 2.8 eV of DOSs as shown in Figure 4b. They are in-plane bonding and antibonding states between Si-C and C-C atoms in c-silicyne, which cause the nearly dispersionless bands in Figure 4a.

For c-germanyne, its flat conformation has a similar electronic property to c-silicyne as shown in the inset of Figure 4c. However, the half-hilled conformation discards the semimetallic feature in c-germanyne, transforming it into a semiconductor as shown in Figure 4c. The valence band maximum (VBM) and conduction band minimum (CBM) are both at the **K** point, which causes a direct bandgap with the PBE value of 0.86 eV. Such gap opening is due to the breaking of sublattice symmetry by the half-hilled buckling in c-germanyne, whose VBM and CBM are located at different Ge atoms as shown in Figure 4e,f. The PDOSs analysis shows that the *p*_*z*_ orbitals of Ge _*u*_ atoms compose the top valence band and the Ge _*f*_*p*_*z*_ ones contribute to the bottom conduction band. As indicated in Figure 2f, the Ge _*u*_ atoms are buckled out of plane, while the Ge _*f*_ ones stay in the same plane with neighbouring C atoms. The corresponding angle of *∠*_*C*−*Ge*−*C*_ is 104° and 120° for the Ge _*u*_ and Ge _*f*_ atoms, respectively. Thus, the hybridization of Ge _*u*_ atom possesses evident *sp*^3^ composition, while the Ge _*f*_ one has a pure *sp*^2^ hybridization. Since the *sp*^3^ hybridization is more favourable than the *sp*^2^ one for Ge element, the *p*_*z*_ orbital of Ge _*u*_ atom is occupied while the Ge _*f*_ one is empty. Therefore, the asymmetric buckling results in two inequivalent Ge atoms, which causes a semiconducting behaviour into c-germanyne.

**Figure 5:**
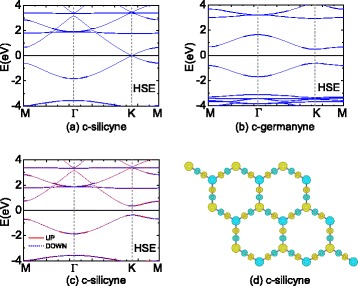
**The HSE band structures of (a) c-silicyne and (b) c-germanyne at the NM state.** **(c,d)** The spin-polarized band structures and spin density distribution for c-silicyne at the AFM state. The isosurface is 0.02 *μ*_*B*_/Å ^3^.

Since the conventional PBE functional would underestimate the bandgaps of semiconductors [54,55], we perform a further calculation by the hybrid HSE XC functional on c-silicyne and c-germanyne. Figure 5 depicts the HSE band structures from the non-spin-polarized calculations, which are analogous to the PBE results in Figure 4. c-Germanyne is found to be a direct-bandgap semiconductor with a sizeable gap of 1.11 eV, and for c-silicyne the semimetallic behaviour is also observed, whose Fermi velocity is increased to 6.4 ×10^5^ m/s by HSE calculations. More interestingly, different from PBE calculations, the spin-polarized HSE calculations find that a spontaneous magnetism would appear in c-silicyne. There is a stable antiferromagnetic (AFM) state, which is 0.014 eV/unit lower than the nonmagnetic (NM) state. The distribution of spin densities for AFM state is shown in Figure 5d. The magnetism of two Si atoms is opposite, which causes a regular anti-parallel coupling between the Si-C and C-C atoms. The Mulliken charge analysis shows the Si and C atoms have a magnetic moment of 0.24 and 0.12 *μ*_*B*_, respectively. Previous studies have reported that the appearance of such AFM state is due to a large ratio of the onsite electron-electron Coulomb energy *U* to the electronic hopping integral *t* on the honeycomb sheet [56]. Thus, for graphene, a large tension is needed to reduce the hopping integral between C *p*_*z*_ orbitals for the antiferromagnetism [56]. Whereas in c-silicyne, the Si *p*_*z*_ orbitals dominate the state around the Fermi level. Due to the existence of -C ≡C- part, the distance between Si atoms is large, which causes a small hopping integral for Si *p*_*z*_ ones. As a result, c-silicyne can possess a stable AFM state without strains. On the other hand, c-germanyne has a gap at the Fermi level, for which the zero density of states hinders the occurrence of spin-polarization. It would be noted that the antiferromagnetism causes a gap opening in c-silicyne at the **K** point as shown in Figure 5c. Following the relativistic dispersion relation of $E(k)=\pm \sqrt {\hbar ^{2} k^{2}{v_{F}^{2}}+m^{2}{v_{F}^{4}}}$ for a massive Fermion [57], the opening gap is related to the Fermion mass as $E_{g}/2=m{v_{F}^{2}}$. Thus, the antiferromagnetism produces a mass for the Dirac fermions in c-silicyne, which is estimated to be 0.15 *m*_0_ from the HSE gap of 0.71 eV (*m*_0_ is the mass of a free electron).

### Strain engineering

**Figure 6:**
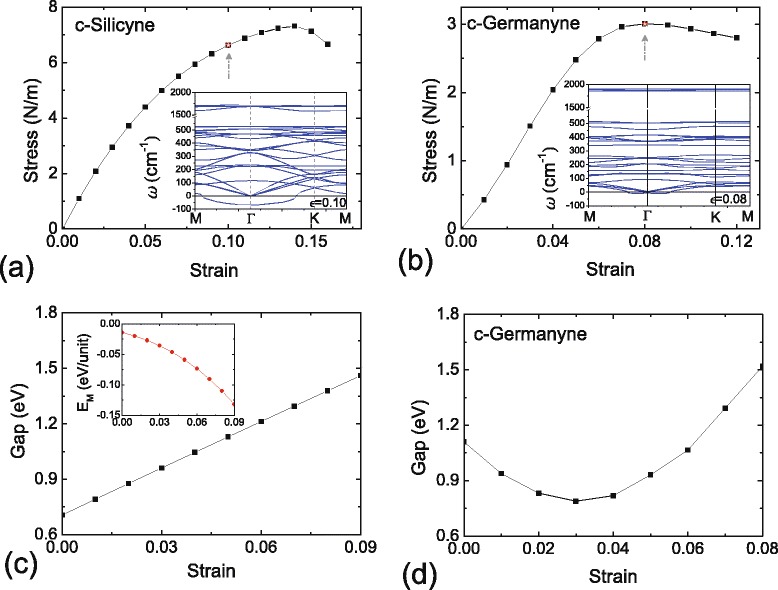
**The stress-strain relation for c-silicyne and c-germanyne.** The inset in **(a)** depicts the phonon curves of c-silicyne at the 0.10 strain, and the inset in **(b)** corresponds to the phonon curves of c-germanyne at the 0.08 strain. **(c,d)** The HSE gap variations of c-silicyne and c-germanyne under the tensile strains. The inset in (c) is the variation of *E*_*M*_ for the antiferromagnetism in c-silicyne.

The strain engineering is an effective way to tailor the properties of nanomaterials [58]. For c-silicyne and c-germanyne, we firstly investigate their elastic characteristics. The elastic moduli are calculated by the energy-vs.-strain method [59,60]. For c-silicyne, we obtain the C _11_ and C _12_ of 61.6 and 54.8 N/m, respectively. Accordingly, the Poisson ratio and Young modulus of c-silicyne are obtained as *ν*=*C*_12_/*C*_11_=0.89 and *E*=*C*_11_−*νC*_12_=12.8 N/m, respectively. While for c-germanyne, its buckled structure weakens the stiffness. The C _11_ and C _12_ drop to 20.2 and 15.0 N/m, respectively, which causes a small value of 9.1 N/m (0.74) for the *E* (*ν*). Comparing to graphyne (C _11_ = 95 and C _12_ = 83 N/m [14], equal to *E*=22.5 N/m and *ν*=0.87), the c-silicyne and c-germanyne sheets are much softer, which would be more easily tailored by external strains. Then, the stress-strain relations of c-silicyne and c-germanyne are investigated by applying homogeneous tensions on the sheets. As shown in Figure 6a, the elastic limit of c-silicyne is at the 0.14 strain with a maximum stress of 7.3 N/m. However, a further check by phonon calculations shows that a soft mode begins to appear at the 0.10 strain, which leads to the in-plane phonon failure for c-silicyne. Thus, the phonon instability reduces the upper bound of strain for c-silicyne, whose critical strain and fracture stress are 0.09 and 6.3 N/m, respectively. While for c-germanyne, its elastic limit is at the 0.08 strain with a maximum stress of 3.0 N/m as shown in Figure 6b. The failure of c-germanyne is determined by the elastic instability, for which under the critical 0.08 strain the stretched sheet is still dynamically stable as shown in the inset of Figure 6b.

Under the strains, an arc-shaped variation of bandgap is found in c-germanyne. As shown in Figure 6d, the tensile strain firstly decreases the gap value, which reaches the minimum of 0.79 eV at the 0.03 strain. Then, the bandgap rises with the increasing strain. It gets to the maximum of 1.52 eV at the critical 0.08 strain. Such non-monotonic variation is attributed to two competitive factors of bandgap in c-germanyne. One is the buckling effect in the structure, which helps the opening of bandgap. While the other is the localization effect induced by strains, which lowers the orbital overlapping. Consequently, the band widths are narrowed and the corresponding bandgap is broadened by the localization effect. Under the strains, the buckles of Ge atoms are weakened, which causes the decrease of bandgap under a small tension. When the strain is increasing, the localization effect becomes more pronounced under the large tension, which increases the bandgap. Thus, the strain-modulated c-germanyne possesses an arc-shaped variation for the bandgap.

For c-silicyne, its NM state is always a semimetal under the strains, similar to the graphene case [61]. While for AFM state, the stability of antiferromagnetism is enhanced under the tensions. The strain-induced localization effect reduces the electronic hopping integral *t*, which leads to a larger value *U*/*t* and avails to a stronger antiferromagnetism. As shown in the inset of Figure 6c, at the 0.02 strain, the magnetic energy (*E*_*M*_=*E*_AFM_−*E*_NM_) of c-silicyne is increased to 0.026 eV/unit, which is approximately the energy of thermal fluctuation at room temperature (*E*_*T*_∼*k*_*B*_*T*=0.0258 eV). When the strain grows up to 0.08 to 0.09, the *E*_*M*_ is even larger than 0.1 eV. It indicates that the AFM state has a robust stability in these strained sheets. Accompanied with the strengthening of AFM state, the bandgap of c-silicyne is also raised. Since the opening gap is dependent on the antiferromagnetism, a monotonic increasing of bandgap is obtained as shown in Figure 6c. The linear relationship exists between the bandgap and tensile strain, and the slope corresponds to a bandgap-deformation potential of 4.39 eV/Å for c-silicyne. At the 0.09 strain, the gap value of c-silicyne can get to 1.46 eV without altering the direct-bandgap feature, suggesting the promising applications for solar energy.

### Solar cell applications

**Figure 7:**
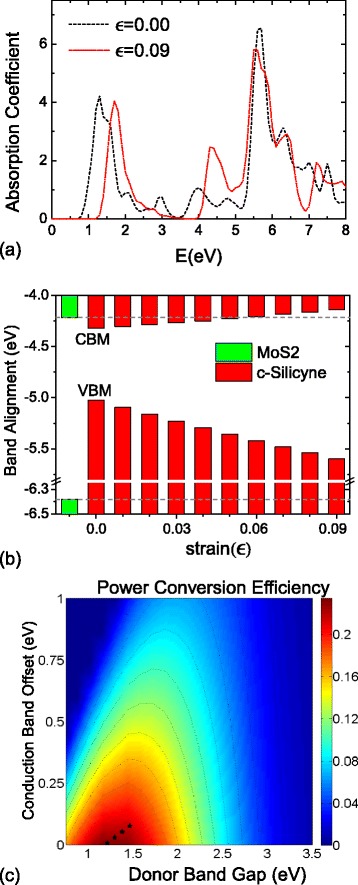
**Optical adsorption, band alignment and power-conversion efficiency.** **(a)** The optical adsorption of c-silicyne at the *ε*=0 and 0.09 strains. **(b)** The band alignment between MoS _2_ and strained c-silicyne sheets. **(c)** Calculated power-conversion efficiency as a function of the donor bandgap and conduction band offset, in which the values of c-silicyne/MoS _2_ system are marked as stars.

The well controllable direct bandgap enables potential solar cell applications for c-silicyne. As a competitive candidate, it requires that c-silicyne has (i) a strong adsorption ability for the visible light and (ii) a suitable band alignment with acceptor materials for the electron-hole separation. To investigate this feasibility, the linear absorption of light is calculated by the HSE XC functional. As shown in Figure 7b, at the strain-free state, due to the interband VBM-CBM transition, a prominent adsorption peak starts from the bandgap of c-silicyne at 0.71 eV. It exhibits a strong adsorption ability up to 3.5 eV. Under tensile strains, the adsorption peak is red-shifted, but it still covers the whole visible-light region of [1.61, 3.10] eV. Thus, c-silicyne meets the first requirement for solar cell applications. Then, the absolute energies of CBM and VBM in c-silicyne are calculated as −4.32 and −5.03 eV, respectively. We find this CBM value is close to that of the MoS _2_ nanosheet, which is −4.22 eV by the same HSE calculations, while the VBM of c-silicyne is much higher than the MoS _2_ value of −6.38 eV. Under the strains, the CBM of c-silicyne moves upwards slowly and it can exceed the MoS _2_ value at the 0.06 strain. The VBM of c-silicyne is down-shifted, but it is always above the VBM of MoS _2_ sheet as shown in Figure 7c. Thus, starting from the 0.06 strain, a type-II band alignment can be formed between the MoS _2_ and c-silicyne sheets, which satisfies the second requirement for high-efficiency solar cells. For c-germanyne, we find that its CBM/VBM is −4.53/−5.64 eV and the strain-induced variation is arc-shaped. Hence, it is hard to find an appropriate common acceptor material for it, which limits the solar energy application of c-germanyne.

When c-silicyne is superimposed with a MoS _2_ sheet, the power conversion efficiency (PCE) *η* for this c-silicyne/MoS _2_ heterostructure can be theoretically estimated in the limit of 100% external quantum efficiency as [62-66]:

$${\fontsize{9}{6} \begin{aligned} \eta=\frac{J_{\text{sc}}V_{\text{oc}}\beta_{\text{FF}}}{P_{\text{solar}}}=\frac{0.65({E_{g}^{d}}-\Delta E_{c}-0.3)\int_{{E_{g}^{d}}}^{\infty}[\!P(\hbar \omega)/\hbar \omega] d(\hbar \omega)}{\int_{0}^{\infty}P(\hbar \omega) d(\hbar \omega)} \end{aligned}} $$

Here, the band-fill factor (*β*_FF_) is adopted to 0.65, the open-circuit voltage (*V*_oc_) is set to ${E_{g}^{d}}-\Delta E_{c}-0.3$, where ${E_{g}^{d}}$ is the bandgap of donor, *ΔE*_*c*_ is the conduction band offset between the acceptor and donor, and 0.3 is an empirical factor for energy conversion kinetics [66,67]. The integral in the numerator is the short circuit current *J*_sc_ calculated in a limit external quantum efficiency of 100% [67,68], and the denominator is the integrated AM 1.5 solar energy flux, which amounts to 1,000 W/m ^2^ [62-66]. We find that under the strains of 0.06 to 0.09, the *η* of c-silicyne/MoS _2_ heterostructure can exceed 20% as shown in Figure 7d. When a 0.06 strain is applied to c-silicyne, the power conversion efficiency *η* reaches the maximum value of 23.1%. Under larger strains of 0.7 to 0.9, *η* becomes 22.5%, 22.4% and 21.4%, respectively. These high power conversion efficiencies are comparable to those of recent proposed phosphorene and graphene-like Si-C nanostructures [62-65]. Therefore, the c-silicyne sheet would be a fascinating nanomaterial for solar energy applications, especially in thin-film photovoltaic systems.

Finally, we briefly discuss the possible synthesis approach for c-silicyne. Benefited from the recent development of metal-assisted coupling methods, several porous nanostructures have been successfully fabricated by self-assembling the molecular building blocks [21,22,69]. Through changing different molecular precursors and metal substrates, the experimenters can design the morphology of novel porous nanosheets and manufacture the well-defined nanostructures [70-74]. It seems the ethynyl(methylene)silyl [75], which contains both Si-C and Si-C ≡C parts in its molecular structure, will be a possible molecular building block for silicyne. After choosing a suitable metal surface as substrates, the metal-catalyzed cross-coupling reaction, which has been used in the synthesis of graphyne [21,22], would be also valid to fabricate the silicyne sheet.

## Conclusions

In summary, we have systematically investigated the structures and properties of porous silicene and germanene nanosheets, i.e. silicyne and germanyne ones. We find that the C-substitution could significantly enhance the energetic and dynamical stabilities of these systems. Different from silicene, the c-silicyne sheet prefers a flat plane, while the c-germanyne favors a unique half-hilled buckled conformation. Such half-hilled conformation brings a semiconducting behaviour into c-germanyne, which is distinct from the graphyne and germanene nanosheets. For c-silicyne, it exhibits a zero-bandgap semimetallic behaviour at the nonmagentic state but becomes a direct-bandgap semiconductor at the antiferromagnetic state. Under tensile strains, the antiferromagnetism in c-silicyne could be strengthened and the bandgap is also linearly raised. More interestingly, when the strained c-silicyne sheet is superimposed onto a MoS _2_ monolayer, the heterostructure can be a promising solar-cell material with high power conversion efficiencies exceeding 20%. Our studies demonstrate that the silicyne and germanyne nanosheets possess peculiar electronic and magnetic properties, which have potential applications in the fields of solar energy and nano-devices.
